# Self-system and mental health status among Malaysian youth attending higher educational institutions: A nationwide cross-sectional study

**DOI:** 10.51866/oa.34l

**Published:** 2024-02-07

**Authors:** Syifa’ Mohd Azlan, Fadzilah Mohamad, Rahima Dahlan, Irmi Zarina Ismail, Hayati Kadir@ Shahar, Khairatul Nainey Kamaruddin, Nur Amirah Shibraumalisi, Sharifah Najwa Syed Mohamad, Nurainul Hana Shamsuddin

**Affiliations:** 1 MBBCh BAO, MMed, Department of Family Medicine, Faculty of Medicine and Health Sciences, Universiti Putra Malaysia, UPM Serdang, Selangor, Malaysia. Email: ilafadzilah@upm.edu.my; 2 BSc Psychology, Department of Family Medicine, Faculty of Medicine and Health Sciences, Universiti Putra Malaysia, UPM Serdang, Selangor, Malaysia.; 3 MBBS, MMed (Psychiatry), Department of Psychiatry, Faculty of Medicine and Health Sciences, Universiti Putra Malaysia, UPM Serdang, Selangor, Malaysia.; 4 MBBS, MMed, Department of Family Medicine, Faculty of Medicine and Health Sciences, Universiti Putra Malaysia, UPM Serdang, Selangor, Malaysia.; 5 MBBCh BAO, MComm Health (Epidemiology & Biostatistic), Department of Community Health, Faculty of Medicine and Health Sciences, Universiti Putra Malaysia, UPM Serdang, Selangor, Malaysia.; 6 MBBCh BAO, MMed Fam Med, Department of Primary Care Medicine, Faculty of Medicine, Universiti Teknologi MARA, Sungai Buloh, Selangor, Malaysia.; 7 MBBS, MMed Fam Med, Department of Primary Care Medicine, Faculty of Medicine, Universiti Teknologi MARA, Sungai Buloh, Selangor, Malaysia.; 8 MBBCh BAO, MMed Fam Med, Department of Primary Health Care, Universiti Sains Islam Malaysia, Persiaran Ilmu, Putra Nilai, Nilai, Negeri Sembilan, Malaysia.; 9 MBBS, MMed, Department of Family Medicine, Faculty of Medicine and Health Sciences, Universiti Putra Malaysia, UPM Serdang, Selangor, Malaysia.

**Keywords:** Depression, Anxiety, Psychological stress, Youth, Mental health

## Abstract

**Introduction:**

Mental health disorders (MHDs) are a global health burden with an increasing prevalence. During the COVID-19 pandemic, depression was the commonest MHD, followed by anxiety and stress. This nationwide study aimed to determine the prevalence and associated factors of depression, anxiety and stress among Malaysian students attending higher educational institutions.

**Methods:**

A cross-sectional study was conducted from June to December 2021. Institutions were selected via stratified random sampling and students via convenience sampling. A self-administered questionnaire comprising questions on socio-demographic characteristics, academic background, substance abuse, childhood abuse, religiosity and the Depression Anxiety Stress Scale-21 was used. Data were analysed using SPSS version 27; descriptive, point-biserial correlation, chi-square and multiple binary logistic regression analyses were conducted.

**Results:**

Sex and adverse childhood experiences significantly predicted all three MHDs (P<0.05). The students from public institutions (odds ratio [OR]=0.71, 95% confidence interval [CI]=0.56—0.90, P=0.004) had a lower risk of depression than those from private institutions. The smokers/vapers (OR=1.43, 95% CI=1.02–2.02, P=0.041) a higher risk of depression than the non-smokers/non-vapers. The social science students (OR=1.29, 95% CI= 1.01–1.65, P=0.039) had a higher risk of anxiety than the science students. The students who highly engaged in organisational religious activity (OR=0.91, 95% CI=0.84–0.98, P=0.015) had a lower risk of anxiety than their counterparts.

**Conclusion:**

Anxiety is the commonest MHD among Malaysian youth, followed by depression and stress. Childhood abuse is a crucial related factor requiring further attention. Screening, surveillance, preventive measures and comprehensive interventions for MHDs should be initiated among youth.

## Introduction

Mental health disorders (MHDs) are a significant global concern with an alarming prevalence. According to the World Health Organization (WHO), MHDs account for 13% of the global burden of health diseases among individuals aged 10–19 years.^1^ In Malaysia, university students have shown a high prevalence of depression (up to 31%), anxiety (up to 60%) and stress (up to 26%).^2,3^ A local study demonstrated a substantial increase in these numbers during the COVID-19 pandemic: 59.2%, 55.1% and 30.6%, respectively.^4^

Previous studies conducted worldwide have shown that psychological distress originates from multiple factors. These factors encompass socio-demographic characteristics such as having a young age, having a female sex, belonging to minority racial and ethnic groups, residing in urban areas, living alone, being single, attending private universities, majoring in science-related fields, having a low household income, having a history of illicit substance use and having parents who are divorced, separated or widowed.^4-13^ Additionally, individual factors such as religiosity^13^ and history of adverse childhood experiences (ACEs), including childhood sexual abuse, physical abuse, emotional abuse and neglect, can significantly impact an individuals mental health status in the long term.^14,15^ A study conducted among a multi-ethnic Asian population has revealed that religiosity, regardless of the specific religion, plays a crucial role in maintaining positive mental health compared with no religion.^16^ This finding corroborates that of a study conducted locally.^13^ According to the WHO, any type of ACE is linked to a 44% increase in the risk of various health conditions in adulthood, including mental illness.^17^ In Malaysia, a similar finding has been reported, wherein about 23% of individuals with a history of ACEs indicated experiencing depression.^18^

To the best of our knowledge, no study has yet been conducted nationwide in this area, particularly among Malaysian youth attending higher educational institutions. This limitation hinders the generalisation of findings. Notably, these stressors may have diflerent effects on diflerent population groups. Understanding these differences is essential for crafting targeted and effective intervention strategies tailored to the local context, particularly during challenging circumstances such as a pandemic. Therefore, this study aimed to investigate the prevalence of MHDs and the association with selfsystem factors, including socio-demographic characteristics, ACEs and religiosity, among Malaysian youth attending higher educational institutions. We hypothesised that there is a high prevalence of MHDs among Malaysian youth attending higher educational institutions, and there is an association between selfsystem factors and MHDs. We utilised the psychodynamic theory by Sigmund Freud as the foundational framework in understanding the association between self-system and psychological distress.^19^ The findings of this study are expected to serve as baseline data for future researchers and relevant authorities towards formulating effective mental health strategies and preventive efforts, with a particular emphasis on self-system.

## Methods

### Study design and setting

This quantitative, nationwide cross-sectional study investigated Malaysian youth attending local higher education institutions. Data were collected from June to December 2021.

### Study population

We recruited students attending higher educational institutions in Malaysia. In particular, Malaysian citizens who were aged 18–24 years and able to understand either the Malay or English language were included. Malaysian citizens who were diagnosed with psychiatric illness were excluded to avoid bias in answering the Depression Anxiety Stress Scale-21 (DASS-21); the tool is meant for screening mental illness.

### Sample size calculation

The sample size was calculated using the singleproportion formula. In a previous study, the prevalence of depression, anxiety and stress was 59.2%, 55.1% and 30.6%, respectively.^4^ With the significance level set at 0.05 and considering an 80% non-response rate for online surveys, the minimum sample size was calculated as 686 participants.

### Sampling method

Two-stage sampling was conducted, with stratified random sampling applied to select institutions and convenience sampling to select students. Stratified sampling was employed to enhance the generalisability of the study to encompass the entire Malaysian tertiary higher-education student population. This approach ensured that each stratum was adequately represented, maintaining proportions accurately. Malaysia was divided into Peninsular Malaysia and East Malaysia, and Peninsular Malaysia was further divided into five regions. Gatekeepers from each selected institution shared the survey link through their official WhatsApp groups as well as official institution emails, and willing students responded to the survey. The number of samples in each region was calculated in proportion to the region’s distribution of students. A total of 32 institutions were selected and approached, but only 25 universities agreed to participate in this study.

### Study instrument

A self-administered online questionnaire, which was available in Malay and English, was used. The first section consisted of questions on selfsystem factors including socio-demographic characteristics, academic background, substance abuse, ACEs and religiosity.

There were four dichotomous questions for ACEs: childhood sexual abuse, physical abuse, emotional abuse and neglect. This scale had an acceptable level of internal consistency, with a Cronbachs alpha value of 0.61. Face validity was tested to check whether the instrument measures what it is supposed to measure. The total score ranges from 0 to 4. Higher scores reflect multiple involvement in ACEs.

The Duke University Religion Index (DUREL) was used to measure the degree to which the participants were involved with religion.^20^ It has a well-established history of usage across diverse cultural and religious contexts, as evidenced by previous research confirming its cross-cultural applicability. The DUREL has high test-retest reliability (intra-class correlation=0.91), high internal consistency (Cronbach’s alpha=0.78-0.91) and high convergent validity with other measures of religiosity (r=0.71-0.86). The Malay-translated version (DUREL-M) also has good internal reliability of 0.8.^21^ It is a Likert scale that consists of five items and measures three dimensions, namely organisational religious activity (ORA), non-organisational religious activity (NORA) and intrinsic religiosity (IR). ORA refers to religious activities performed in a formal and public setting; NORA refers to religious activities performed privately; and IR refers to performing religious activities as an ultimate end.^22^ The total score ranges from 5 to 27. Higher scores reflect a high degree of religiosity.

The second section of the questionnaire was the DASS-21. The scale is a validated selfreported screening tool used to assess three domains, namely depression, anxiety and stress. The depression domain measures feelings of hopelessness, devaluation of life, anhedonia and lack of interest. The anxiety domain assesses autonomic arousal, skeletal muscle effects, situational anxiety and the presence of anxious feelings. The stress domain assesses chronic, non-overreactive arousal. The DASS-21 has high reliability scores, with a Cronbach’s alpha value of 0.91 for the depression domain, 0.84 for the anxiety domain and 0.90 for the stress domain.^23^ The Malay version of the DASS-21 was adapted from a previous study. This version also has good Cronbach’s alpha values of 0.84 for the depression domain, 0.74 for the anxiety domain and 0.79 for the stress domain.^24^ It uses a 4-point Likert scale, and each domain consists of seven questions. The sum score is calculated separately for each domain. The total score is multiplied by two and categorised into normal, mild, moderate, severe and extremely severe. In this study, the scores were dichotomised into normal and abnormal (mild to extremely severe). The cut-off scores for normal were ≤9 for depression, ≤7 for anxiety and ≤14 for stress.

### Data collection

Students were approached by gatekeepers from each institution. The gatekeepers were members of the Student Representative Council and Student Affairs Department. A link to the informed consent form was given via email and WhatsApp, and students who met the inclusion criteria were provided a subsequent link to the questionnaire. The survey was conducted anonymously and confidentially, encouraging honest responses and reducing social desirability bias to ensure genuine responses without the fear of being criticised or judged.

### Data analysis

Data were managed and analysed using SPSS version 27 (IBM, Chicago, IL). They were cleaned to detect missing values, coding errors or illogical values. Descriptive statistics were computed for all variables. Categorical data were reported as frequencies and percentages and continuous data as means and standard deviations (SDs). The association between the variables was evaluated using chi-square analysis, and the predictors of depression, anxiety and stress were determined using multiple binary logistic regression analysis. The significance level was set at P<0.05.

### Ethical approval

Ethical approval was obtained from the Medical Research and Ethics Committee for Research Involving Human Subjects at Universiti Putra Malaysia (approval ID: JKEUPM-2021-141). The data obtained were kept confidential and will be destroyed after 5 years. The respondents who scored severe or extremely severe levels in the DASS-21 domains were contacted for further assessment when they consented.

## Results

A total of 1211 students from public and private higher educational institutions (universities and university colleges) across Malaysia responded to the online survey. Of them, 40 (3%) did not fulfil the inclusion criteria: Eight refused to participate further in this study; five were currently under follow-up for psychiatric illness; 25 were not from the selected institutions (non-short-listed institutions - the questionnaire link could have been shared by the target respondents); and two respondents had incomplete data, all of whom were thus excluded. The final useable data were obtained from 1171 students, and the total response rate was 99.3%.

### Participant characteristics

The mean age of the participants was 20.16 (SD 1.66) years. More than half of the participants were aged 18-20 years (60.3%, n=706), suggesting that most of them were transitioning into adulthood and thus facing unique challenges and stressors. Most participants were women (70%, n=820), raising the possibility of a potentially higher prevalence of MHDs, as women are often recognised as being more vulnerable to certain stressors than men. Almost two-thirds of the participants were Malays and Muslims (66.5%, n=779), providing a cultural context that could influence the outcomes. Most participants (89.2%, n=1044) stayed with their families during the data collection, which may indicate that most students had a strong support system. Over half of the participants lived in urban areas (65.8%, n=770). Only 0.3% (n=3) were married, and a small proportion (15.6%, n=183) reported that their parents were either divorced/separated or widowed. Over half (58.5%, n=685) reported that they came from the below 40% group with a household income of RM 4849 and below. Approximately 15.5% had a smoking or vaping history; 17.8% consumed alcohol; and 0.9% admitted to having a history of drug abuse. These factors may have contributed significantly to the mental health burden experienced by the participants. The socio-demographic characteristics are summarised in Table 1

**Table 1:** Participant characteristics.

Variables	n	%
Age		
18–20 years	706	60.3
21–24 years	465	39.7
Sex		
Male	351	30.0
Female	820	70.0
Race		
Malay	730	62.3
Non-Malay	441	37.7
Religion		
Muslim	779	66.5
Non-Muslim	392	33.5
Locality		
Rural	401	34.2
Urban	770	65.8
Living arrangement		
With family	1044	89.2
Without family	127	10.8
Current relationship status		
Single/in a relationship	1168	99.7
Married	3	0.3
Parents’ marital status		
Married	988	84.4
Others	183	15.6
Household income		
≤RM 4849	685	58.5
RM 4850-10959	357	30.5
≥RM 10960	129	11.0
Academic background Institution		
Public	589	50.3
Private	582	49.7
Field of study		
Science	449	38.3
Social science	722	61.7
Year of study		
Year 1	531	45.3
Year 2	307	26.2
Year 3	228	19.5
Year 4	105	9.0
Substance abuse Smoking history		
Yes	181	15.5
No	990	84.5
Alcohol intake		
Yes	209	17.8
No	962	82.2
Drug abuse		
Yes	10	0.9
No	1161	99.1
Adverse childhood experiences	0.22	0.61^*^
Religiosity		
Organisational religious activity	4.82	1.57^*^
Non-organisational religious activity	4.24	1.96^*^
Intrinsic religiosity	12.73	2.23^*^

* Mean (SD)

### Academic background

Among the respondents, the majority (45.3%, n=531) were first-year students, and only a small proportion (9.0%, n=105) were in their final year. A larger proportion of the respondents were social science students, and only 38.3% (n=449) were science students. The distribution of the respondents studying at private and public universities/colleges was almost equal: 50.3%, n=589 vs 49.7%, n=582. These findings indicated that most students were in their adjustment period to university life, varying in academic background, stressor and exposure to science-related knowledge.

### ACEs

The mean number of ACEs among the respondents was relatively low (0.22, SD=0.614). Approximately 2.7% experienced childhood sexual abuse; 4.3%, physical abuse; 11.3%, emotional abuse; and 4.3%, neglect.

The existence of these ACEs, even among a small proportion of the participants, could result in enduring adverse effects on the mental health of these individuals during their adulthood.

### Religiosity

The religiosity varied between the three types, namely ORA, NORA and IR; the means and SDs were 4.82 (1.57), 4.24 (1.96) and 12.73 (3.23), respectively. This variation in religiosity may play role in shaping the participants’ belief systems and engagement with religion, which could influence their mental health outcomes.

### Prevalence of depression, anxiety and stress

Approximately 45.6% (n=534) of the participants reported having depressive symptoms; 60.5% (n=709), anxiety symptoms; and 40% (n=468), stress symptoms (Table 2).

**Table 2:** Prevalence of depressive, anxiety and stress symptoms among the Malaysian youth attending higher educational institutions (N=1171).

Variables	n	%
Depressive symptoms		
Normal (≤9)	637	54.4
Abnormal (>9)	534	45.6
Anxiety symptoms		
Normal (≤7)	462	39.5
Abnormal (>7)	709	60.5
Stress symptoms		
Normal (≤14)	703	60.0
Abnormal (>14)	468	40.0

### Associated factors of depression, anxiety and stress

Table 3 shows the inferential analysis of the factors associated with depression, anxiety and stress. Sex, parents’ marital status and ACEs were significantly associated with depression, anxiety and stress (P<0.05). Conversely, the type of institution, field of study and three types of religiosity were significantly associated only with depression and anxiety. A history of smoking and vaping was significantly associated with depression alone and age with anxiety alone. The variables that were significant (P<0.05) in the bivariate analysis were further analysed and included in the multiple binary logistic regression analysis.

**Table 3:** Associated factors (self-system) of the mental health status among the Malaysian youth attending higher educational institutions (N=1171).

Variables	Depressive symptoms			Anxiety symptoms			Stress symptoms		
	Normal	Abnormal	P	Normal	Abnormal	P	Normal	Abnormal	P
Age 18-20 years 21-24 years	370 (52.4) 267 (57.4	336 (57.4) 37.1 (42.6)	0.092	262 (37.1) 200 (43.0)	444 (62.9) 265 (57.0)	0.043^*^	479 (67.8) 323 (69.5)	227 (32.2) 142 (30.5)	0.560
Sex Male Female	216 (61.5) 421 (51.3)	135 (38.5) 399 (48.7)	0.001^*^	164 (46.7) 298 (36.3)	187 (26.4) 522 (63.7)	<0.001^*^	275 (78.3) 527 (68.5)	76 (21.7) 293 (31.5)	<0.001^*^
Race Malay Non-Malay	397 (54.4) 240 (54.4)	333 (62.4) 201 (45.6)	0.990	298 (40.8) 164 (37.2)	432 (59.2) 277 (62.8)	0.218	506 (69.3) 296 (67.1)	224 (30.7) 145 (32.9)	0.433
Religion Muslim Non-Muslim	420 (53.9) 217(55.4)	359 (46.1) 175 (44.6)	0.64	308 (39.5) 154 (39.3)	471 (60.5) 238 (60.7)	0.934	532 (68.3) 270 (68.9)	247 (31.7) 122 (31.1)	0.839
Locality Rural Urban	223 (55.5) 414(53.8)	179 (44.5) 355 (46.2)	0.593	159 (39.6) 303 (39.4)	243 (60.4) 466 (60.6)	0.960	276 (68.7) 526 (68.4)	126 (31.3) 243 (31.6)	0.929
Living arrangement With family Without family	573 (54.1) 64(57.1)	486 (45.9) 48 (42.9)	0.540	423 (39.9) 39 (34.8)	636 (60.1) 73 (65.2)	0.292	724 (68.4) 78 (69.6)	335 (31.6) 34 (30.4)	0.782
Current relationship status Single/in a Relationship Married	635 (54.4) 2 (66.7)	533 (45.6) 1 (33.3)	0.669	460 (39.4) 2 (66.7)	708 (60.6) 1 (33.3)	0.334	799 (68.4) 3 (100.0)	369 (31.5) 0 (0.00)	0.239
Parents’ marital status Married Others	553 (56.0) 84 (45.9)	435 (44.0) 99 (54.1)	0.012^*^	402 (40.7) 60 (32.8)	586 (59.3) 123 (67.2)	0.045^*^	689 (69.7) 113(61.7)	299 (30.3) 70 (38.5)	0.033^*^
Household income ≤RM 4849 RM 4850-10959 ≥RM 10960	373 (54.5) 192 (53.9) 72 (55.4)	312(45.5) 164(46.1) 58 (44.6)	0.959	264 (38.5) 137 (38.5) 61 (46.9)	421 (61.5) 219(61.5) 69 (53.1)	0.181	466 (68.0) 242 (68.0) 94 (72.3)	219(32.0) 114(32.0) 36 (27.7)	0.610
Institution Public Private	344 (59.2) 293 (49.7)	237 (40.8) 297 (50.3)	0.001^*^	211 (35.8) 251 (43.2)	379 (64.2) 330 (56.8)	0.009^*^	389 (65.9) 413(71.1)	201 (34.1) 168 (28.9)	0.058
Field of study Science Social	266 (59.1) 371 (51.5)	184 (40.9) 350 (48.5)	0.011^*^	199 (44.2) 263 (36.5)	251 (55.8) 458 (63.5)	0.008“	315(70.0) 487 (67.5)	135 (30.0) 234 (32.5)	0.379
Year of study Year 1 Year 2 Year 3 Year 4	289 (54.5) 163 (53.1) 125 (54.6) 60(57.1)	241 (45.5) 144 (46.9) 104 (45.4) 45(42.9)	0.911	215(40.6) 112(36.5) 90 (39.3) 45 (42.9)	315(59.4) 195 (63.5) 139 (60.7) 60 (57.1)	0.589	370(69.8) 200 (65.1) 161 (70.3) 71 (67.6)	160 (31.2) 107 (34.9) 68 (29.7) 34 (32.4)	0.493
Substance abuse Smoking/vaping history Yes No	82(45.3) 555(56.1)	99 (54.7) 435 (43.9)	0.008^*^	64 (35.4) 398 (34.0)	117 (64.6) 592 (59.8)	0.220	113(62.4) 689 (69.6)	68 (37.6) 301 (30.4)	0.056
Alcohol intake Yes No	103 (49.3) 534 (55.5)	106 (50.7) 428 (44.5)	0.101	390 (40.5) 72 (34.4)	572 (59.5) 137 (65.6)	0.102	134 (64.1) 668 (69.4)	75 (31.5) 294 (30.6)	0.133
Drug abuse Yes No	3 (30.0) 634 (54.6)	7 (70.0) 527 (45.4)	0.120	3 (30.0) 459 (39.5)	7 (70.0) 702 (60.5)	0.539	4 (40.0) 798 (68.7)	6 (60.0) 363 (31.3)	0.051
Adverse childhood experiences^#^			<0.001^*^			<0.001^*^			<0.001^*^
Religiosity Organisational religious activity^#^ Non-organisational religious activity^#^ Intrinsic religiosity^*^			0.071^*^ 0.034^*^ 0.006“			0.012^*^ 0.026^*^ 0.017^*^			0.328 0.510 0.220

# Point-biserial correlation test

§ Pearson chi-square test

* Significant (P<0.05)

### Predictive model for depression, anxiety and stress

Multiple logistic regression analysis was conducted to determine the predictors of depression, anxiety and stress, and the findings are shown in Table 4. Sex and ACEs significantly predicted the development of depression, anxiety and stress. The female students had a 1.59, 1.45 and 2.12 times higher risk of depressive, anxiety and stress symptoms, respectively, than the male students. The risk of depressive symptoms among the students who had a history of smoking or vaping was 1.43 times higher than that among the students who had no such history. Two other predictors of the development of anxiety symptoms were identified: field of study and ORA. The social science students were 1.29 times more likely to have anxiety symptoms than the science students. In terms of religiosity, only high participation in ORA was found to be significant as a protective factor against anxiety symptoms (adjusted odds ratio=0.91, P=0.005) during the COVID-19 pandemic. Conversely, the students studying at public universities were 0.71 less likely to have depressive symptoms than those studying at private universities.

**Table 4:** Predictive model for depression, anxiety and stress (N=1171).

Factors	B	SE	AOR	95% CI		P
				Lower	Upper	
**Depression**						
Sex Male Female	0.64	0.14	1.59	1.22	2.07	<0.001^*^
Parents’ marital status Married Others	0.29	0.17	1.33	0.96	1.85	0.085
Institution Private Public	-0.34	0.12	0.71	0.56	0.90	0.004^*^
Smoking/vaping history No Yes	0.36	0.18	1.43	1.02	2.02	0.041^*^
Adverse childhood experiences	0.48	0.11	1.61	1.30	2.00	<0.001^*^
**Anxiety**						
Sex Male Female	0.37	0.13	1.45	1.12	1.88	0.005^*^
Age 18–20 years 20–24 years	-0.22	0.12	0.81	0.63	1.03	0.084
Field of study Science Social science	0.26	0.13	1.29	1.01	1.65	0.039^*^
Adverse childhood experiences	0.44	0.12	1.55	1.23	1.95	<0.001^*^
Religiosity: Organisational religious activity	-0.10	0.04	0.91	0.84	0.98	0.015^*^
**Stress**						
Sex Male Female	0.75	0.16	2.12	1.57	2.87	<0.001^*^
Smoking/vaping history No Yes	0.32	0.18	1.38	0.96	1.98	0.079
Adverse childhood experiences	0.52	0.10	1.68	1.37	2.07	<0.001^*^

* Significant (P<0.05); B, regression coefficient; SE, standard error; AOR, adjusted odds ratio; CI, confidence interval. All assumptions were met. Neither extreme outliers nor multicollinearity was present. The goodness-of-fit of the model was checked using the omnibus test of model coefficients.

## Discussion

This study aimed to determine the prevalence of MHDs among Malaysian youth and the factors contributing to these conditions. The present findings corroborate previous reports among university students in the United States and in Selangor during the pandemic, where the majority of university students reported experiencing moderate-to-extremely severe levels of anxiety symptoms.^25,26^ Both studies reported a high prevalence of psychological distress, with more than 50% exceeding clinical cut-off points. However, a prior study conducted before the pandemic showed a lower prevalence of psychological distress among university students, with only 21% reporting depression, 50% experiencing anxiety and 12% reporting stress.^27^ Such disparities in the reported prevalence could be attributed to the impact of COVID-19 and different phases of the outbreak. The significant rise during the earlier phase of the COVID-19 pandemic could be attributed to overwhelming feelings during the lockdown, as students were confined at home and had to adjust their lifestyles considerably. In contrast, the changes during the later phases could be attributed to an accumulation of apprehensive feelings stemming from the beginning of the outbreak, negatively affecting the quality of life. However, all previous studies have shared a common similarity wherein women reported higher levels of moderate-to-extremely severe mental health issues, as also noted in this study. Women tend to cope with psychological distress through rumination, constantly thinking about unpleasant emotions and the reason behind those emotions.^28^ This prolonged exposure to negative emotions may eventually contribute to a greater likelihood of female students experiencing psychological distress than male students particularly during the outbreak.

Herein, the students studying at public institutions were less likely to experience depressive symptoms than those studying at private institutions. This contradicts previous reports that students studying at public institutions are more prone to depression owing to the burden of heavier academic assignments and often coming from lower-income households.^29^ Conversely, students studying at private institutions face significant psychological distress, attributed to factors such as online classes, high tuition fees and extended semesters incurring additional fees.^12^ These challenges could have had a more pronounced impact on students during the pandemic, potentially deteriorating their mental health. Additionally, our study found that the social science students were more prone to experiencing anxiety than the science students. This finding may be explained by that of a study conducted in Jordan, which confirmed that science students possessed a higher degree of knowledge and exposure to general information related to the pandemic and were more likely to outsource information from reliable scientific sources.^10^ Therefore, science students may have better understood what to expect during the pandemic and been better able to regulate themselves.

A history of abusive childhood experiences and engagement in substance abuse showed an association with psychological distress in this study. The adverse long-term effects of unpleasant childhood events may persist into adulthood owing to the traumatic, toxic and stressful experiences during their most vulnerable period of life. The accumulation of these negative emotions may permanently alter brain development, resulting in poor mental health.^15^ Moreover, students who have experienced multiple childhood adversities are more likely to develop mental health problems than those who have experienced fewer or no adversities.^14^ Similarly, nicotine in cigarette/e-cigarette could alter brain functioning by elevating mood through the release of dopamine (positive hormone). However, this unnatural release of dopamine temporarily relaxes users while reducing the brain’s capacity to produce dopamine naturally.^30^ This could result in them smoking more for the pleasing effect and in a greater dependency on nicotine, increasing the likelihood of severe depression and stress.^8^ Nevertheless, our study found no association between smoking and anxiety levels, which corroborates previous evidence.^8^

Concerning religiosity, the students who engaged in ORA were less likely to experience anxiety. However, this finding contradicts a previous report among adults in Selangor, in which there is no association between anxiety and ORA.^13^ Students participating in ORA are more likely to participate in public religious activities, giving them opportunities to interact with religious communities. This exposure may motivate individuals to become more consistent in religious practice, potentially easing anxiety symptoms.^13^

### Strengths

This study was a nationwide study that investigated the prevalence of depression, anxiety and stress among youth in Malaysia, providing insights into the national prevalence among the population. In addition to the socio-demographic characteristics, the study identified other factors that have not been previously explored such as religiosity.

### Limitations and recommendations

The main limitation of this study is that the data were collected from students selected using non-probability convenience sampling, limiting the external validity. The survey link was distributed via WhatsApp, leading to its unintended circulation among individuals who were not part of the intended target sample. However, we addressed this issue by subsequently removing these cases from our dataset. Additionally, the online data collection limited the direct contact between the respondents and researchers, which could have hindered the respondents from seeking clarifications about the questionnaire. Nevertheless, the online questionnaire enabled the exploration of sensitive issues, such as child abuse, and minimised the risk of disease transmission during the COVID-19 pandemic. Future researchers are recommended to employ probability sampling and conduct face-to-face data collection wherein researchers are readily available for inquiries.

## Conclusion

Anxiety is the most common MHD among Malaysian youth attending higher educational institutions. There is a considerable increase in the prevalence compared with prior reports. Sex is significantly associated with depression, anxiety and stress, while smoking/vaping history is the only significant predictor of depression. The academic background or institution is significantly associated with depression, whereas the field of study is significantly associated with anxiety. The presence of ACEs is related to higher risks of depression, anxiety and stress, while involvement in ORA is associated with a lower risk of anxiety.

## Data sharing statement

All information obtained in this study will be kept confidential and will not be made publicly available to the extent permitted by law.

**How does this paper make a difference in general practice?**

- The findings could serve as baseline data on the prevalence of depression, anxiety and stress and the associated factors among Malaysian youth.
- The study highlights that the current leading mental health disorder among Malaysian youth is anxiety.
- Sex and adverse childhood experiences are the most significant predictors of mental health disorders among Malaysian youth.
